# From spasms to smiles: how facial recognition and tracking can quantify hemifacial spasm severity and predict treatment outcomes

**DOI:** 10.1007/s00701-024-06407-1

**Published:** 2025-01-07

**Authors:** Ahmed Al Menabbawy, Lennart Ruhser, Ehab El Refaee, Martin E. Weidemeier, Marc Matthes, Henry W. S. Schroeder

**Affiliations:** 1 Department of Neurosurgery, University Medicine Greifswald, Greifswald, Germany; 2 Department of Neurosurgery, Cairo University, Giza, Egypt

**Keywords:** Hemifacial spasm, Facial recognition, Facial tracking, Microvascular decompression, Classification system

## Abstract

**Purpose:**

Currently available grading and classification systems for hemifacial spasm either rely on subjective assessments or are excessively intricate. Here, we make use of facial recognition and facial tracking technologies towards accurately grouping patients according to severity and characteristics of the spasms.

**Methods:**

A retrospective review of our prospectively maintained preoperative videos database for hemifacial spasm was done. Videos were analyzed using an Apple AR kit-based App. A facial mesh is automatically allocated to specific biometric facial points. Videos are analyzed using Blender software for measuring the amplitude and frequency of the spasms. Classification of the patients into groups was done using both divisive k-means and agglomerative hierarchical clustering. Correlation-Analysis with preoperative quality of Life (Qol) using SF-36 questionnaire and HFS-8 score was performed. Additionally, correlation with postoperative outcome was calculated.

**Results:**

79 preoperative videos were included. Both up-bottom and bottom-up clustering approaches grouped the patients into 3 different clusters according to 4 variables (eye closure, mouth distance change, rate, and repetition of the spasms). Correlation of the groups with the Qol was done for 46/79 patients (58.2%). Spasms could be classified into mild, moderate clonic and severe tonic spasms. Patients with mild spasms showed better Qol scores. Moderate clonic spasms experienced best outcomes following microvascular decompression.

**Conclusion:**

This novel classification using facial-tracking and augmented-reality is easy to use and apply. It quantifies the severity and type of the spasms and relates it to the quality of life of patients, postoperative outcome, and could guide our management strategy.

## Introduction

Hemifacial spasm involves involuntary contractions usually located on one half of the face due to a vascular compression at the nerve exit zone [9, 14, 15]. Few classification and grading systems have been introduced to classify hemifacial spasms (HFS) aiming to aid in treatment decisions and outcome monitoring. However, existing grading systems often suffer from complexity and overlapping categories, hindering implementation [16, 21]. Consequently, patient self-assessment remains crucial in evaluating symptom improvement or deterioration. In the age of technological advancements, tools like facial recognition and tracking technology offer promising solutions that can be used to aid in classification and grading process [21, 1, 11]. Current grading systems have tried to grade hemifacial spasms in an objective manner as an attempt to guide the management decision and monitor the outcome. The most popularly known grading systems are the symptomatic change grading system (SMC), the hemifacial spasm grading scale (HSGS) and the HFS-7 and HFS-8 questionnaires [11]. 

Microvascular decompression (MVD) is the treatment modality of choice when complete cure of the spasms is aimed. However, the question is: are all spasms the same regarding nature and severity? And the second question: would these patients all benefit the same way from surgery, Botox injection, or medical treatment?

To overcome the limitations in the current classifications and provide answers to the aforementioned questions, we underwent this study to utilize facial recognition and facial tracking technologies to quantify, grade, and classify the hemifacial spasms, facilitating a comprehensive assessment of their impact on treatment outcomes objectively and accurately.

## Methods

Patients written consents were obtained preoperatively for using the data and videos for research purposes. This study was designed and implemented in accordance with “Standards for Reporting of Diagnostic Accuracy” (STARD) guideline statement [4].

Retrospective review of preoperatively recorded patients’ videos was done. Videos of patients with a confirmed diagnosis of hemifacial spasm from our MVD database from January 2017 till December 2022 were extracted and analyzed. We intended to include only preoperative videos of patients with evident microvascular compression syndrome from MRI Constructive Interference in Steady State Magnetic Resonance Imaging (CISS) and Time of Flight (TOF) sequences, within 7 days before surgery date. Each included video should be of high quality and contain the whole face of the patient with a minimum of 15 s of spasms. Videos of patients with facial palsy due to botulinum toxin injection or with other movement disorders or non-confirmed compression syndrome on MR imaging were excluded. All videos were recorded within 7 days before surgery.

### Establishing the measurement parameters

**Fig. 1:**
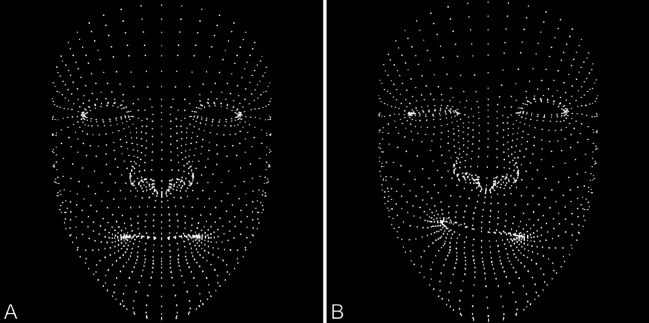
Showing the automatic plotting of the “facial points mesh” using the AR-kit facial tracking tool. Left side (**A**) shows resting position and right (**B**) shows spastic position

We used the open-source AR-facial tracking kit from Apple (Apple Inc., USA) in the form of an IOS Application and "Blender" software [12, 13, 17]. This kit can capture the surface and dimensions of areas and objects without getting disturbed by minor changes in lighting, camera, or object movement and is highly sensitive. Afterwards it projects and creates a 2D-Model which can be used for subsequent analysis. In this manner, a "Face Mesh" is created, projecting a cloud of points/vectors at the patient’s face and extracting the face structure in this way, detecting the motion of each point/vector (Fig. 1).

**Fig. 2:**
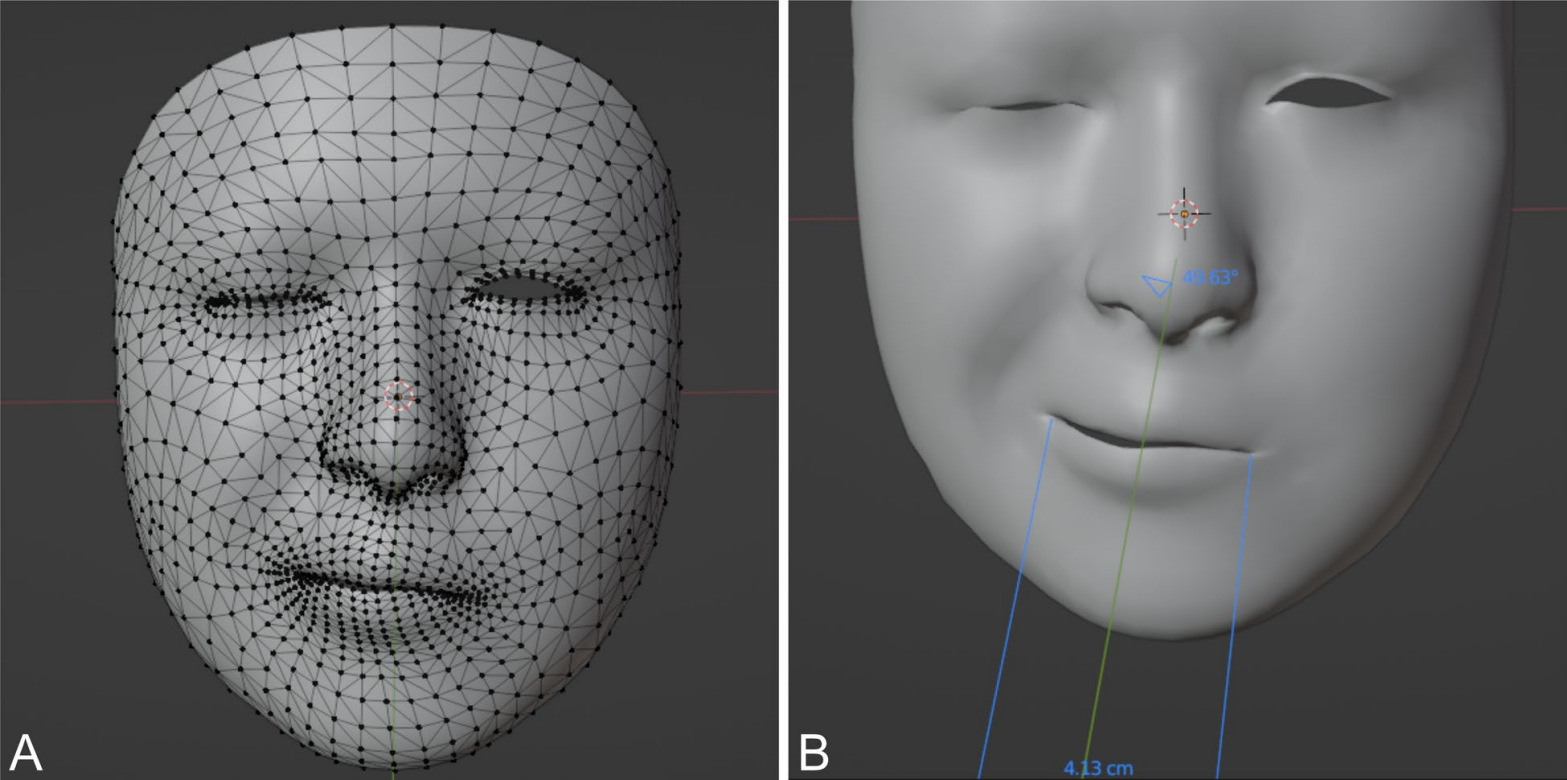
Showing a screenshot from “Blender software” while analyzing the facial movements using the already established facial points mesh. **A**: Importing of the face mesh into Blendar with all vector points. **B**: Shows how the distance between angles of the mouth is measured

The Mesh is then uploaded to the software “Blender” (Fig. 2A) [13]. Within “Blender” we used the measuring tool of the program to connect the predefined distances and measured them within the dynamics of the video (Fig. 2B). The maximum and minimum of the distance was used to calculate the percentage change. Frequency of the spasms can be measured through calculating the time frames in which the spasms take place per second. There are two main parameters for a contraction/spasm: the amplitude and frequency of the spasms.

### Spasm amplitude parameters

**Fig. 3:**
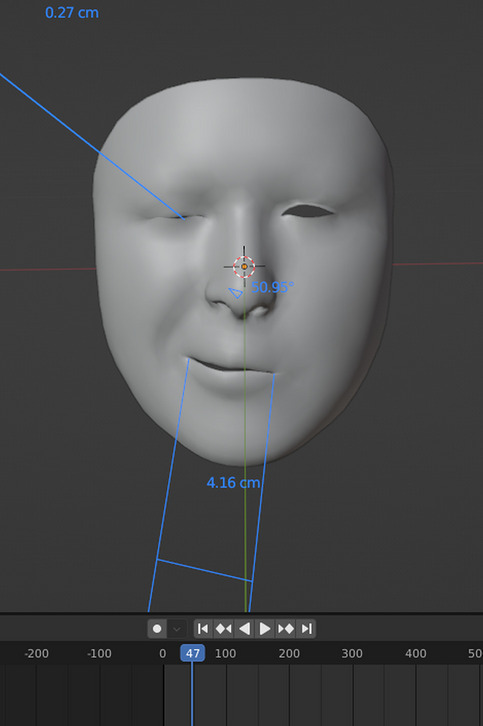
Shows how also the eye closure distance is measured. The change in the distance was calculated and divided by the resting state distance to normalize/standardize the results for all patients and the percentage change % was used for the analysis. Measurement of the change in the distance between the angles of the mouth is also illustrated

To standardize the parameters for amplitude our aim was setting predefined and reproducible points of the face that can be applied to all patients. To prevent mistakes of individual face forms and sizes, we used the percentage changes in the predefined distances. Two parameters were used to evaluate the main muscle groups involved in the spasms. Firstly, the percentage change in the distance between both angles of the mouth. Secondly, the percentage change in the eye closure (Figs. 2B and 3).

The angle of the mouth distance was measured as the distance traveled by the vector at the point of maximum contraction. The eye closure percentage was measured in comparison to the resting state of the other eye when the spasms were not present and in cases of clonic contractions in comparison to the resting state of the contralateral eye. A complete eye closure was considered 100% contraction and whenever the eye was not involved this was considered 0% amplitude.

### Spasms frequency parameters

#### Rate of the spasms

This was determined using the time frames on Blender and calculated as spasm/second. The videos were taken that one second is represented over 20 frames and accordingly was the rate calculated.

### Repetition/frequency of the spasms

This was obtained from the patients in a form of questionnaire as one of 4 categories in descending order of intensity: permanent spasms episodes (Frequeny 4), 1–5 min between each episode (Frequency 3), 5–10 min between episodes (Frequency 2) and ten minutes or more between episodes (Frequency 1).

### Clustering method

First, basic descriptive statistics to describe the patients’ demographic characteristics were employed. Two types of clustering methods were implemented: (a) hierarchical agglomerative bottom-up approach, with three linkage criteria (minimum or single-linkage; maximum or complete-linkage; and average linkage); (b) divisive hierarchical clustering up-bottom approach using k-means and elbow method. The metric to measure the dissimilarity between the sets of values was the Euclidean distance between the set of points. R software and R-Studio were used for this part [25].

### Correlation with quality of life

Correlation with preoperative Quality of Life (Qol) was done through two Qol questionnaire results; HFS-8 questionnaire and the short form 36 questionnaire (SF-36). Analysis was performed using one way Analysis of variance (ANOVA) test, P-value considered significant when < 0·05.

### Correlation with Outcome following Microvascular decompression

Furthermore, correlation with the patient reported outcome after microvascular decompression was performed both immediately (5 days following MVD) and on the longterm (at last reported follow-up with minimal follow-up of 12 months). Patients with cure or improvements of Spasms more than 90% were considered to have good outcomes. The correlation analysis was performed using one way ANOVA and P-value considered significance when < 0·05.

## Results

96 videos met our inclusion criteria. For these videos only data from 79 patients (where daily frequency of repetition of the spasms could be obtained) were complete. 60·8% of the patients were females (48/79). Mean age of the patients (SD) was 54·5(11·7) years at the date of the operation with a mean follow-up (SD) of 28(14·7) months.

### Clustering results

**Fig. 4:**
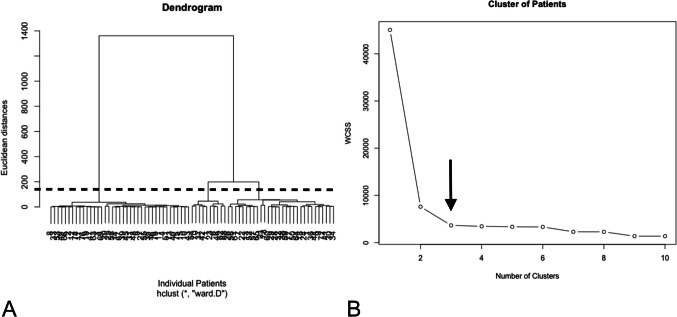
Showing both the dendrogram method to determine the number of clusters (**A**) and the elbow method to determine number of clusters (**B**). Arrow shows the elbow and dashed black line shows the cut point of the dendrogram. Both methods resulted in 3 clusters

**Fig. 5:**
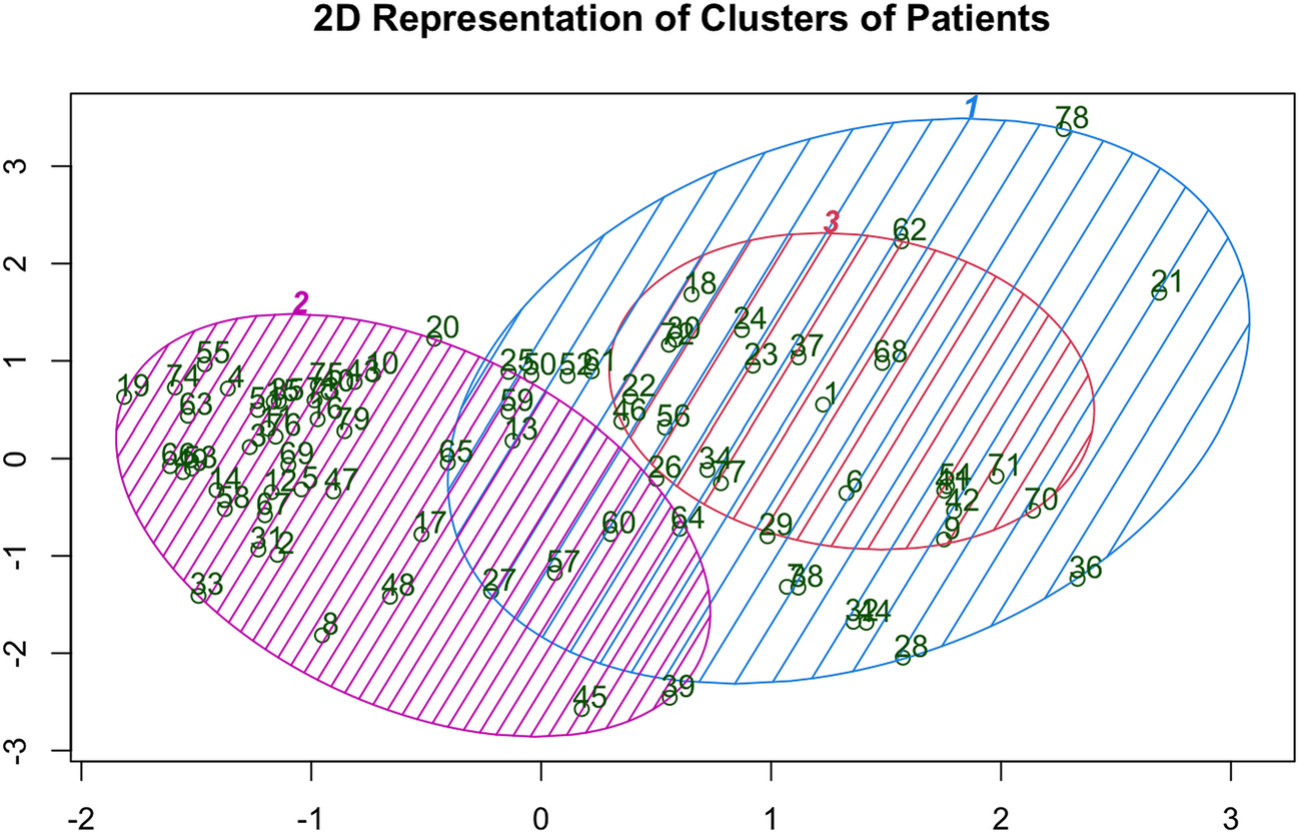
Two-dimensional demonstration of the 3 clusters on the x and y axes. Dimension one (x-axis) is the change in the distance between angles of the mouth and dimension 2 is the change in eye closure (y-axis). Areas of overlap witnessed because of the two other non-demonstrated dimensions. The three ovals represent the three clusets and corresponding patient number

Both hierarchical and divisive clustering approaches clustered the patients into three different clusters according to the 4 investigated factors (percentage change in the distance between mouth angles, percentage of eye closure, rate of the spasms (spasm/second) and patient reported frequency of repetition of the Spasms). This is illustrated in Fig. 4 according to the dendrogram and elbow method. The three clusters are illustrated in Fig. 5.

The Within cluster’s sum of squares (WCSS) was as follows; Cluster 1: 408·3657, Cluster 2: 961·8343 and Cluster 3: 2276·7942. The proportion of variance was 91·9% explained by the clustering (SS/ totalSS = 91·9%).

Both agglomerative and divisive clustering resulted in the same number of patients and even same individual patients within clusters in both methods which means 100% identical classification. Cluster 1 contained 39 patients (49·4%), cluster 2 had 10 patients (12·7%) and cluster 3 clustered 30 patients (37·9%).

The values of cluster-means for the K-means analysis are tabulated in Table 1. From these values we observe that patients in Cluster 1 had their eyes almost spared from contraction, rate of the spasms was the lowest. This led us to name this group the “mild spasms”.

**Table 1:** Showing the cluster means according to k-means and follow-up periods

Cluster number	Mouth angles distance change %	Eye closure change (%)	Rate of spasms (spasm/second)	Repetition of spasms (Grade 1–4)	Average FU period in months (SD)
1 mild 39/79	3.743317	0	0.4223077	3.051282	25.3 (16.7)
2 moderate clonic 10/79	7.065610	39.47300	0.7250000	3.600000	20.4 (14.7)
3 severe tonic 30/79	8.349610	62.38033	0.6738333	2.866667	33.0 (17.3)

Cluster 2 characteristics were that they had the fastest average rates and repetition of the spasms (more frequent spasms) with moderate eye closure and moderate change in the angle of the mouth. This led us to name this group the “moderate clonic spasms”.

Cluster 3 characteristics were as follows; largest affection of both amplitude parameters and medium rate of contraction so we call it the “severe tonic spasms”.

### Correlation of the clusters with quality of life

The SF-36 scores were calculated and standardized to the values of the German population. SF-36 preoperative questionnaires were only available for 46 patients. The mean physical and mental summary scores of Qol SF-36 for each cluster are given in Table 2. Both total physical and mental summary scores were highest among Group 1 (mild Spasms) which means better Qol in comparison to the two other groups. The mental summary score showed gradual worsening with worsening of the spasms’ severity (Table 2). However, the moderate clonic spasms group showed the worst physical summary score among the three groups. HFS-8 scores were only available for 46 patients and also showed better total scores for patients with mild spasms in comparison to the other two groups as seen in Table 2. These differences, however, did not reach statistical significance using ANOVA.

**Table 2:** Showing qol results of SF- Questionnaire, HFS-8 score, immediate and long-term outcomes following microvascular decompression

Cluster number	Physical summary score (standardized to German population)	Mental summary score (standardized to German population)	HFS-8 average score	Outcome after MVD	
				Immediate improvement (% of total patients)	Long-term improvement (% of total patients)
1 mild spams	55.06	36.83	11.9545455	87.18%	84.61%
2 moderate Clonic	48.91	35.46	15.1428571	90%	100%
3 severe Tonic	52.96	34.33	15.3529412	83.33%	86.67%
F value	1.90219	0.1459	1.35102	0.67634	1.09614
P Value	0.161589	0.864667	0.269747	0.511506	0.339384

SF-score of the normal population score is 50. Above 50 means higher Qol score and less than 50 means lower score than the mean population score

### Correlation with outcome following MVD

As seen in Tables 2 and 90% of patients with moderate clonic spasms (Group 2) improved immediately after surgery in comparison to 87·2% and 83·3% for both mild and severe spasms group respectively. On the other hand, long term improvement was witnessed in all patients (100%) of moderate clonic spasms in comparison to 84·6% in the mild spasms group and 86·7% in the severe tonic spasms group.

## Discussion

### Main annotations of the study

The key finding of the study is the establishment of an accurate and user-friendly grading and classification system for hemifacial spasm that precisely allocates the spasms type into simply one of three severity groups (mild spasms group, moderate clonic spasms group, and severe tonic spasms group). The overall preliminary results from these first 79 patients suggest that the clustering has managed to group the spasms effectively, with relatively small within-cluster variations and significant separation between the clusters. According to the results, the clustering explains approximately 91·9% of the total variance, which is considered a very good result.

The method’s mathematical and algorithmic presentation may seem complex and challenging in usability. However, our current efforts involve creating a user-friendly desktop and mobile App, derived from our findings, enabling patients to conveniently access it whenever they experience the spasms and even providing them with individual predictions about post management course and outcomes and comparing the spasms state before and after treatment.

Secondly, the new classification is associated with Qol where mild spasms are associated with better scores of the SF-36 and HFS-8. Thirdly, patients with clonic spasms (moderate clonic spasms group) showed best outcome following MVD.

### The new classification and quality of life

Although all patients with HFS showed generally SF-36 average scores lower than the general population, patients with mild spasms showed better scores both on the mental summary and physical summary scales which means that the severity of the spasms affect the quality of life of patients as well and not that all patients with HFS are the same regarding Qol [2, 27]. However, these results did not reach statistical significance due to the small number of the patients available (46 patients). Similarly, the HFS-8 score was lower in the mild Spasms group in comparison to the moderate and severe groups.

On the Mental aspect of Qol the severe tonic spasms showed the worst outcome which means the more severe the spasms are, the more is the mental and psychological burden on the patients. Nevertheless, physical aspects of Qol showed different results where the clonic spasm seemed to hinder physical Qol more than even severe tonic contractions. We used HFS-8 rather than HFS-7 because it is the updated version and includes sleep quality [3]. 

Other studies utilized SF-36 questionnaires for correlation of patients’ Qol before and after treatment such as after botulinum toxin injection or MVD. Others used them to compare HFS patients to control groups. Such studies showed clear statistically significant results but did not concentrate on the differences between the severity of Spasms among patients’ different groups like we did here in our study. We believe that after adopting this accurate classification system and more verification of our results, statistical significance can be reached [10, 22, 24]. 

### Can Spasm Severity affect the outcome of therapy?

Since our center is a referral center for microvascular decompression [1, 2, 5–8, 18, 19], correlation of the spasms’ severity and outcomes of microvascular decompression (MVD) both on the short and long term is a very important question. Although all patients from the three groups benefited from MVD, this analysis has shown that cluster 2 (moderate clonic spasms) was the group that had best outcomes both on short and long term. This is quite interesting; however, it would be more interesting to test also the response to botulinum toxin Therapy regarding the different grades/clusters. However, generally MVD is so far the only curative treatment option [20]. That is why more studies should be conducted on this new classification with other therapy options as it might play a vital role in changing the management recommendation plans for HFS patients.

### Comparison to other grading systems

The two most popular HFS grading systems are the symptomatic change grading system (SMC) and the HFS-7 questionnaire [11]. The former is mainly based on the physicians and patients’ evaluation which makes it somehow carry more objectivity. On the other hand, the latter is solely based on the patient’s subjective evaluation of the Spasms which carries a bias possibility. For instance, according to the SMC classification, if the spasms are only involving the periocular muscles the spasms are graded as lowest grade. However, and according to our grading system this would mean worse Qol for such patients whenever the eyes are involved. The new classification system almost eliminates this subjectivity. Moreover, the SMC classified patients into 4 different grades and the HFS-7 questionnaire can result in any number from 0 to 28 [27]. However, our grading system concluded that the best classification would be a 3-group classification.

The hemifacial spasm grading scale (HSGS) is another grading system that has been introduced and respects similar aspects which our grading system also respects including localization, frequency and intensity of the spasms [11, 23]. However, in the HSGS, the patients were asked whether the spasms were present more or less than 50% of the time. In our cohort, all patients reported that the spasms occurred more than 50% of the time and stated that the spasms were almost the whole time there or they had some minutes between every spasm attack and the next one [11]. Moreover, it was always considered in the localization whether it affected the eye alone or both eye and mouth. But affecting the mouth alone was not an option for the patients adding to the limitations of the HSGS grading system together with its subjectivity.

Regarding the handiness of the classification system, our new classification would be the easiest to establish because the patient has only to take a video of his/her face while the spasms are present and answer just one question (How often the spasms occur daily? ).

### Availability for the patients

We are planning the development of a Mobile App available for all patients with HFS. The App would not only grade and classify the spasms, but also compare the spasms severity before and after treatment whether following botulinum toxin injection or MVD or any other treatment modality (including Botox therapy) giving the patient immediate feedback at home about the response to treatment.

### Limitations of the study and future research

The study has several limitations worth noting. Firstly, and, despite achieving a highly accurate mathematical clustering of the 79 patients, only data of 46 patients were available to evaluate for correlation with Qol and HFS-8. Moreover, a larger dataset would be beneficial for future assessments of the grading system’s effectiveness in managing the condition. Lastly, while the patients were clustered into 3 groups and some correlations to the quality of life could be concluded, these conclusions did not show statistical significance due to the small sample size. Severity of the spasms might be correlated to the severity of the compression or other factors. This should be also studied as possible factors for difference in spasms severity but this is out of the scope of this study and is planned for future studies [26]. 

## Conclusion

Our utilization of facial recognition and tracking technology has facilitated the development of a precise classification system for hemifacial spasm, effectively categorizing the spasms into three distinct groups: mild, moderate clonic and severe tonic.

This grading system could provide some correlation with the patients’ quality of life. By offering this novel approach, our grading system seeks to guide treatment decisions and efficiently monitor outcomes, presenting a promising resolution to the challenges posed by existing grading systems. Moreover, it allows for an objective comparison of preoperative and postoperative outcomes, enhancing our understanding of treatment efficacy. And when the development of the mobile App. is available, this tool will be available for every patient diagnosed with hemifacial spasm. In conclusion, our study highlights the potential benefits of this innovative approach in advancing the assessment and management of hemifacial spasm.

## Data Availability

No datasets were generated or analysed during the current study.
